# Perceptual and locomotor factors affect obstacle avoidance in persons with visuospatial neglect

**DOI:** 10.1186/1743-0003-11-38

**Published:** 2014-03-19

**Authors:** Gayatri Aravind, Anouk Lamontagne

**Affiliations:** 1 School of Physical & Occupational Therapy, McGill University, Montreal, QC, Canada; 2 Feil and Oberfeld Research Centre, Jewish Rehabilitation Hospital, Research cite of CRIR, 3205 Place Alton Goldbloom, Laval, Quebec, Canada

**Keywords:** Circumvention, Collisions, Hemineglect, Perception, Stroke, Virtual reality, Walking

## Abstract

**Background:**

For safe ambulation in the community, detection and avoidance of static and moving obstacles is necessary. Such abilities may be compromised by the presence of visuospatial neglect (VSN), especially when the obstacles are present in the neglected, i.e. contralesional field.

**Methods:**

Twelve participants with VSN were tested in a virtual environment (VE) for their ability to a) detect moving obstacles (perceptuo-motor task) using a joystick with their non-paretic hand, and b) avoid collision (locomotor task) with moving obstacles while walking in the VE. The responses of the participants to obstacles approaching on the contralesional side and from head-on were compared to those during ipsilesional approaches.

**Results:**

Up to 67 percent of participants (8 out of 12) collided with either contralesional or head-on obstacles or both. Delay in detection (perceptuo-motor task) and execution of avoidance strategies, and smaller distances from obstacles (locomotor task) were observed for colliders compared to non-colliders. Participants’ performance on the locomotor task was not explained by clinical measures of VSN but slower walkers displayed fewer collisions.

**Conclusion:**

Persons with VSN are at the risk of colliding with dynamic obstacles approaching from the contralesional side and from head-on. Locomotor-specific assessments of navigational abilities are needed to appreciate the recovery achieved or challenges faced by persons with VSN.

## Introduction

Visuospatial neglect (VSN) is an attentional-perceptual disorder affecting 25% to 30% of persons living with the consequences of a stroke [1,2]. It alters the detection and utilisation of relevant visual information from the side opposite to the brain lesion [3]. It is best described as a failure to report, respond to or orient to novel or meaningful stimuli presented to the side opposite the brain lesion [4]. VSN has been shown to impact motor performance in a variety of tasks [5,6], including locomotion [7,8]. While independent walking is one of the main goals of rehabilitation post stroke [9], persons with VSN demonstrate a poor walking recovery [10]. They show deviations in their walking trajectory [11], collide with walls and furniture [12] and present with an increased risk of falls [13,14], making independent walking unsafe [15]. Attentional bias to the ipsilesional side due to lack of inhibition by the affected hemisphere [16], distorted space representations [17] and alack of visual exploration on the contralesional side [18] have been suggested as explanations for their colliding behaviours.

Community ambulation involves challenges of different terrains and entities that may enter into one’s walking path [19]. Dynamic obstacles, which are commonly encountered in community environments such as malls and crowded streets, are especially challenging in that they have constantly changing spatio-temporal characteristics. Avoidance of dynamic obstacles demands the retrieval and processing of information obtained from the environment as well as the planned and coordinated execution of online locomotor adjustments [20]. This requires simultaneous and coordinated functioning of attentional, sensory and motor systems, which can be compromised in post-stroke VSN. To our knowledge, the ability of persons with VSN to negotiate dynamic obstacles while walking remains unexplored but is highly pertinent to rehabilitation of such individuals [21]. In a recent report, participants who were apparently completely recovered from VSN based on standard ‘paper and pencil’ assessments were shown to display altered walking trajectory adjustments in response to changing visual motion information [22]. It was suggested that clinical assessments might not be adequate to identify deficits in processing visual motion and far space stimuli. Furthermore, a complex and challenging task such as walking may lead to the neglect symptoms becoming more apparent [23]. These observations raise the question as to whether conventional clinical assessments for VSN can explain functional performance while walking. In this study, we examined the ability of persons with VSN to detect moving obstacles (perceptuo-motor task) and to avoid collisions with such obstacles during a goal directed locomotor task performed in a virtual environment (VE). The VE provided the ideal setting given that it is safe, controlled and ecological while yielding behaviours similar to what is observed in the real world [24]. We hypothesized that in individuals with VSN, the abilities to detect and circumvent moving obstacles approaching from the neglected (contralesional) side are altered as compared to the non-neglected (ipsilesional) side. We further hypothesized that the performance in the perceptuo-motor task better explains obstacle avoidance behaviours while walking than that on clinical VSN assessments.

## Methods

Sample size was estimated using GPower 3.1.2, for the *analysis of variance for repeated measures* with the 3 directions of approach as a “within” subject factor, assuming a large effect size (0.40) and moderate correlation (0.50) between directions of obstacle approaches. A sample size of 12 participants was obtained at a power of 80% and a type 1 error of 0.05.

Twelve participants with VSN following a first time unilateral supratentorial stroke (Table 1) were recruited from an inpatient rehabilitation centre based on the following inclusion criteria: a stroke confirmed by a CT scan/MRI; a clinical diagnosis of VSN based on the motor free visual perceptual test (MVPT) and/or the Star Cancellation test; an ability to walk independently with or without a walking aid over 10 metres; and motor recovery scores ranging from 3 to 6 out of 7 on the leg and foot impairment inventories of the Chedoke McMaster Stroke Assessment. Individuals with a diagnosed visual field defect (Goldman perimetry test), cognitive deficits (scores <26 on the Mini-Mental State Examination) or other co-morbid conditions (musculo-skeletal, cardiovascular, neurologic) interfering with locomotion were excluded. Participants varied in their comfortable walking speed with values ranging from 0.45 to 1.02 m/s (0.74 ± 0.17 m/s, mean ± 1SD). Six of them used a cane during the experiment. The study was approved by the ethics committee of the Centre for Interdisciplinary Research in Rehabilitation of Greater Montreal. All participants gave their informed consent to participate in the study and to publish the resulting data and patient details.

**Table 1:** Participant characteristics

**Participant**	**Age (yrs)**	**Gender**	**Cane**	**VSN (MVPT +)**	**MoCA (/30)**	**CMMSA leg, foot (/7)**	**Chronicity (months)**	**Etiology of CVA**	**Site of lesion**	**Collisions**
1	50	F	N	+	28	6,5	90	Ischemic	Unspecified right MCA supplied territories	CL + M
2	63	M	Y	+	28	4,3	10	Hemorrhagic	Right subcortical regions, internal capsule, thalamus	M
3	67	F	N	+	23	5,3	6	Ischemic	Unspecified right subcortical regions	None
4	52	F	N	+	27	4,3	4	Ischemic	Right temporo-parietal, frontal	None
5	57	M	Y	+	23	4,3	5	Hemorrhagic	Left internal capsule	M
6	57	F	Y	+^*^	25	5,3	7	Ischemic	Unspecified right MCA supplied territories	None
7	57	M	N	+	27	5,4	4	Ischemic	Right internal capsule, thalamus & basal ganglia	CL + M
8	72	F	Y	+	-^†^	4,3	6	Ischemic	Left MCA supplied territories	None
9	65	M	N	+	24	6,4	10	Ischemic	Right MCA supplied territories, watershed areas of ACA and MCA	CL + M
10	72	F	N	+	24	5,4	3	Ischemic	Right Internal capsule, Posterior parietal area with diffuse cerebral atrophy	CL + M
11	69	F	Y	+	24	4,3	13	Ischemic	Right MCA including temporal areas, corona radiata, grey nucleus	M
12	47	F	Y	+	28	5,5	4	Ischemic	Unspecified right MCA supplied territories	CL + M

*Only Cancellation Test was reported in the chart; † Could not assess due to language barriers.

*Abbreviations: VSN*, Visuospatial neglect; *MVPT*, Motor Free Visual Perceptual Test; *MoCA*, Montreal Cognitive Assessment; *CMMSA*, Chedoke-McMaster Stroke Assessment (CMMSA); *MCA*, Middle cerebral artery; *CVA*, cerebrovascular accident, *ACA*, Anterior cerebral artery; *M*, middle; *CL*, contralesional; *SD*, standard deviation.

### Experimental set-up and procedures

Participants took part in two evaluation sessions taking place no more than one week apart and which included, in a random order, clinical tests, the perceptuo-motor task and the locomotor task. Clinical assessment comprised tests for visuospatial neglect (Bells [25] and Line Bisection [26] tests), cognitive/executive function (Montreal Cognitive Assessment, Trail Making B) and comfortable walking capacity over 10 m. All participants were identified as right handed on the Edinburg Handedness Inventory.

**Figure 1:**
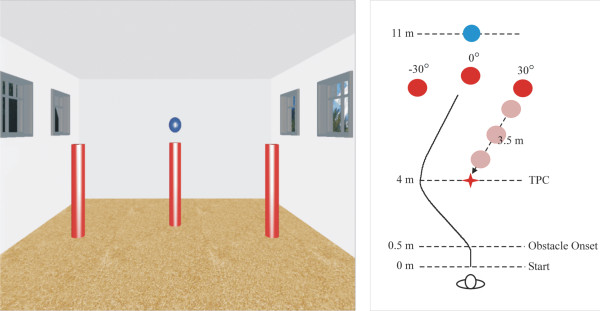
**Schematic representation of the virtual scene.** The left panel shows the screenshot of the virtual scene with the 3 red cylindrical obstacles and the blue target. The right panel illustrates the dimensions of the testing area and the relative positions of the participant and the obstacles. The red star symbol represents the theoretical point of collision (TPC).

The perceptuo-motor and locomotor tasks were conducted while the participants viewed the VE in an nVisor SX60 head mounted display (HMD) (NVIS, USA). The VE consisted of a room with dimensions matching that of the physical room (12 m × 8 m). A blue circular target was present on the wall at the far end (11 m) of the virtual room and three red cylinders (obstacles) were positioned in front of a theoretical point of collision in an arc of radius 3.5 m at 0° (middle) and 30° right and left (Figure 1). The theoretical point of collision (TPC) is the point where the participant and the obstacle paths, if left unaltered, would meet and collide together. Participants were positioned at the beginning of the virtual room facing the centred target. After advancing forward by 0.5 m, one of the 3 obstacles randomly started moving in the direction of the TPC and beyond at a speed of 0.75 m/s. A fixed speed and a fixed angle of approach was chosen in order to keep the walking distance to the target consistent. The diagonal obstacles crossed the midline (straight path from starting position to the target) after crossing the TPC.

### Locomotor task

In the locomotor task, participants were instructed to walk at comfortable speed towards the blue target. They were instructed to avoid a collision with an approaching obstacle, if any, but were not given any instructions on how to avoid the obstacle. A trial could present one of the four conditions randomly: obstacle approaching from the centre, left or right, as well as a control trial which was devoid of any moving obstacle. Control trials were used to determine baseline walking speed and trajectory in the absence of moving obstacles. In case of a collision, visual feedback was provided in the form of a flashing “Collision” sign. Participants were provided with 2 practice trials per condition and 4 to 7 trials per condition were collected, depending on endurance.

Positions of 3 reflective markers placed on the HMD were tracked by a 12-camera Vicon-512™ motion capture system (UK) and fed to the CAREN 3™ virtual reality software (Motek BV, Amsterdam) to provide the real-time update of the participants’ perceived position and orientation in the VE. Markers were also placed on specific body landmarks specified in the full body maker set of the Plug in Gait model from Vicon, with 2 additional markers placed on the walking aid when applicable. Data were recorded at 100Hz in CAREN 3™ and at 120Hz in Vicon™.

### Perceptuo-motor task

For the perceptuo-motor task, participants were seated and responded using a joystick (Attack3, Logitech, USA) held by the non-paretic hand and placed at a comfortable height, while viewing the VE in the HMD. The forward motion of the obstacle was set at 0.75 m/s, a speed representative of ambulatory stroke population [27]. A forward displacement of 0.5 m triggered one of the 3 obstacles to move, or a catch trial with no moving obstacle. The catch trials were aimed at preventing anticipatory responses. The participants were instructed to press the joystick button as soon as they perceived the onset of obstacle motion, or to withhold any response in the absence of an obstacle. In the failure to press the button in the presence of a moving obstacle, the obstacle continued to move ahead and a collision ensued. The participant was not informed about the collision event. Participants were provided 2 practice trials for each condition and performed 10 trials for each of the 4 conditions for a total of 40 conditions.

### Data analysis

For the purpose of the analyses, obstacles were identified as approaching from the contralesional side, the middle and the ipsilesional side. For the perceptuo-motor task, the detection time was calculated as the time taken after the movement onset of the obstacle for the subject to press the button. For the locomotor task, the minimum absolute distance was calculated as the minimum distance maintained between the participant and the obstacle, before the obstacle passed beyond the participant. The number of trials in which a collision was detected was divided by the total number of trials for each of the conditions to give the percent collision. In order to determine the presence of a collision, a critical distance was set for each participant, calculated as the sum of the radius of the obstacle and the distance between C7 and the lateral-most marker on the body or walking aid. When the distance between the participants and the obstacle dropped below this critical distance, a collision event was detected. Onset of an avoidance strategy was measured as the time at which a medio-lateral displacement (of the head markers) exceeding 0.25 m (half of average shoulder width) on either side was detected. Preferred sides of avoidance strategy were also noted.

### Statistical analysis

The effects of direction of obstacle approach (i.e. contralesional, head on, ipsilesional) on detection time, minimum absolute distance and onset of avoidance strategy were examined using separate repeated measure analyses of variance (ANOVAs), followed by Tukey post-hoc comparisons with Bonferroni adjustments. Probability level was set at p < 0.05. Collision rates were compared across conditions using a non-parametric Kruskall-Wallis test. Pearson correlation coefficients were used to quantify the relationship between measures of obstacle avoidance performance (minimum distance, percent collision, onset of avoidance strategy) and performance on the perceptuo-motor task (detection time) as well as on clinical assessment of neglect (Bell’s Test, Line Bisection Test), executive function (Trail Making B) and walking capacity (walking speed). Correlations were carried out separately for each obstacle approach.

## Results

### VSN and Perceptuo-motor performance

**Figure 2:**
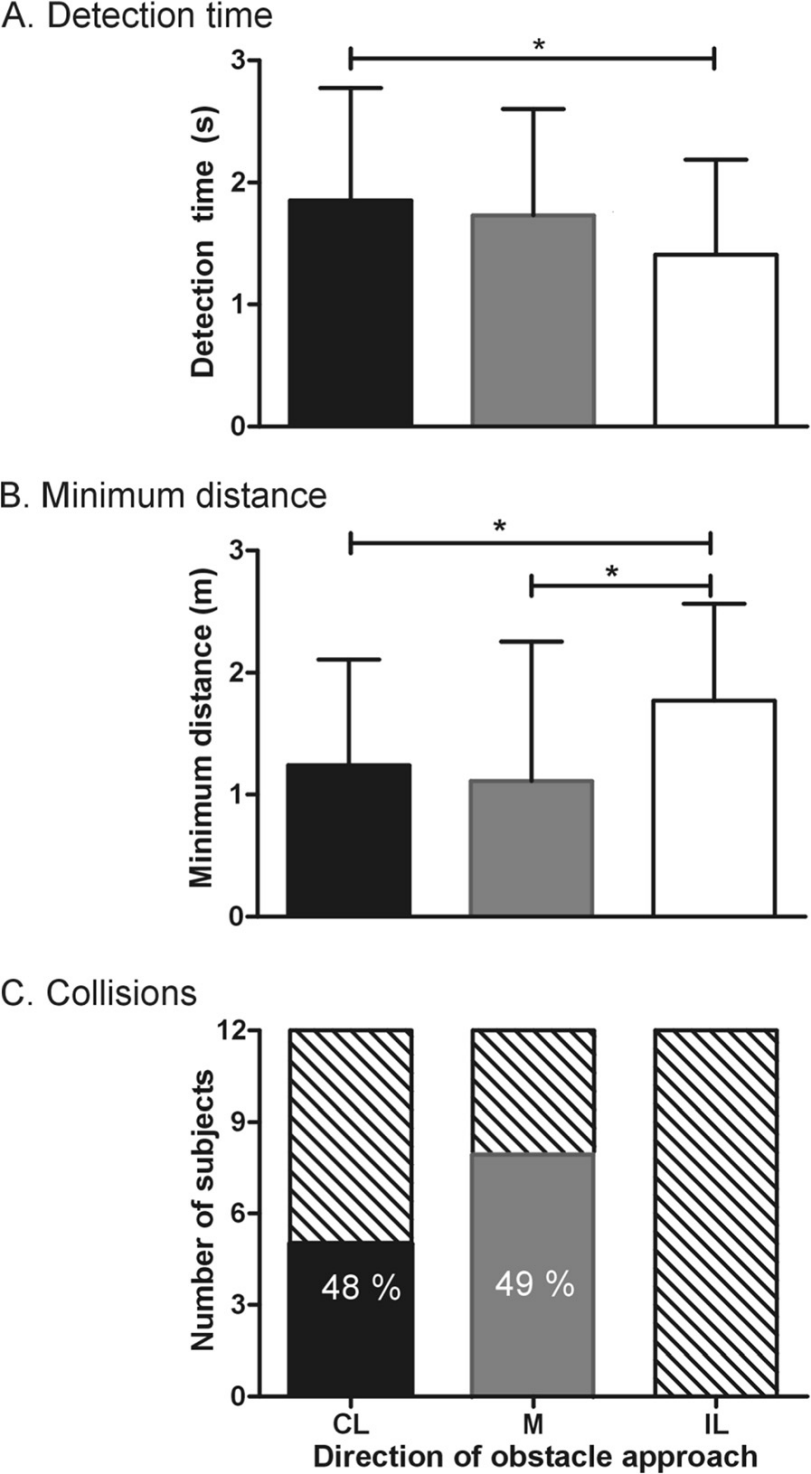
**Mean ± 1SD values of all participants for A) detection times [perceptuo-motor task] B) Minimum absolute distances maintained between the participants and the obstaclesas well as C) the distribution of colliders (solid) and non-colliders (lined) [locomotor task] for the contralesional (CL), middle (M) and ipsilesional (IL) obstacles.** The digits in the solid columns indicate the average percentage of trials in which collisions were recorded for the specific obstacle direction. * p < 0.05.

Presence of VNS was confirmed in all 12 participants, with positive results on the Bells and/or Line Bisection tests. Participants scoring positive (>6 omissions [25]) on the Bell’s test (n = 4), showed 6 to 18 omissions. Those positive (error >0.6 cm [26]) on the line bisection test (n = 12), showed errors between 0.9 and 4.8 cm. On the perceptuo-motor task, there was a significant difference in detection times across directions (F(3, 35) =20.72; p = 0.01) with participants taking significantly longer times (p < 0.05) to detect contralesional than ipsilesional obstacles (Figure 2A).

### Locomotor performance

**Figure 3:**
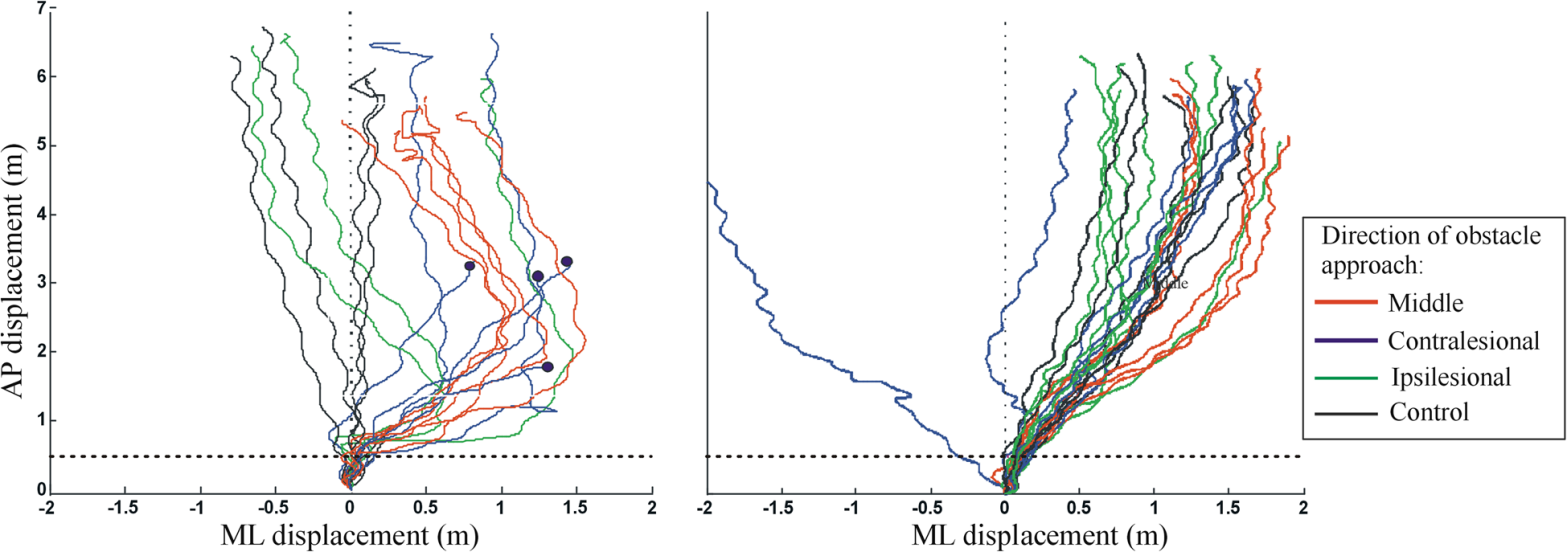
**Representative diagram of walking strategies adopted by 2 participants.** The left panel is the walking pattern of a collider while the right panel consist of walking pattern of a non-collider. Note the collisions experienced by participant #12 [# omissions on Bells test = 9] (see left panel), which are represented by the black dots, and the absence of collision for participant #6 [# omissions on Bells test = 0] (see right panel).

No falls occurred during the testing and none of the participants reported any discomfort or dizziness due to the VE. Figure 3 represents walking trajectories of two participants, one collider and one non-collider, in response to different obstacle approaches. Both participants showed a clear preference to deviate to the ipsilesional side, sometimes even in the absence of an obstacle i.e. in the control trials (see non-collider). The collider participant repeatedly collided with the contralesional obstacle, which caused him to stop walking, and showed no collision for the middle and ipsilesional obstacles.

When considering all participants, the minimum distance maintained from the obstacle differed across obstacle directions [F(3, 35) = 8.133; p = 0.0114]. Compared to the ipsilesional obstacle, participants maintained smaller distances from the contralesional (p < 0.005) and middle obstacles (p < 0.05) (Figure 2B). This difference was maintained when the collision trials were excluded from the analysis (F(3, 35) = 9.159; p = 0.001). Five participants out of 12 collided with the contralesional obstacle and 8 collided with the middle obstacle, while no collisions occurred with the ipsilesional obstacle in any of the participants. Average percent collisions were 48.11% (12% to 70% of trials) and 49.34% (40% to 65% of trials), respectively, for participants showing collisions with the contralesional and middle obstacles (Figure 3C).

### Colliders vs. non-colliders

**Figure 4:**
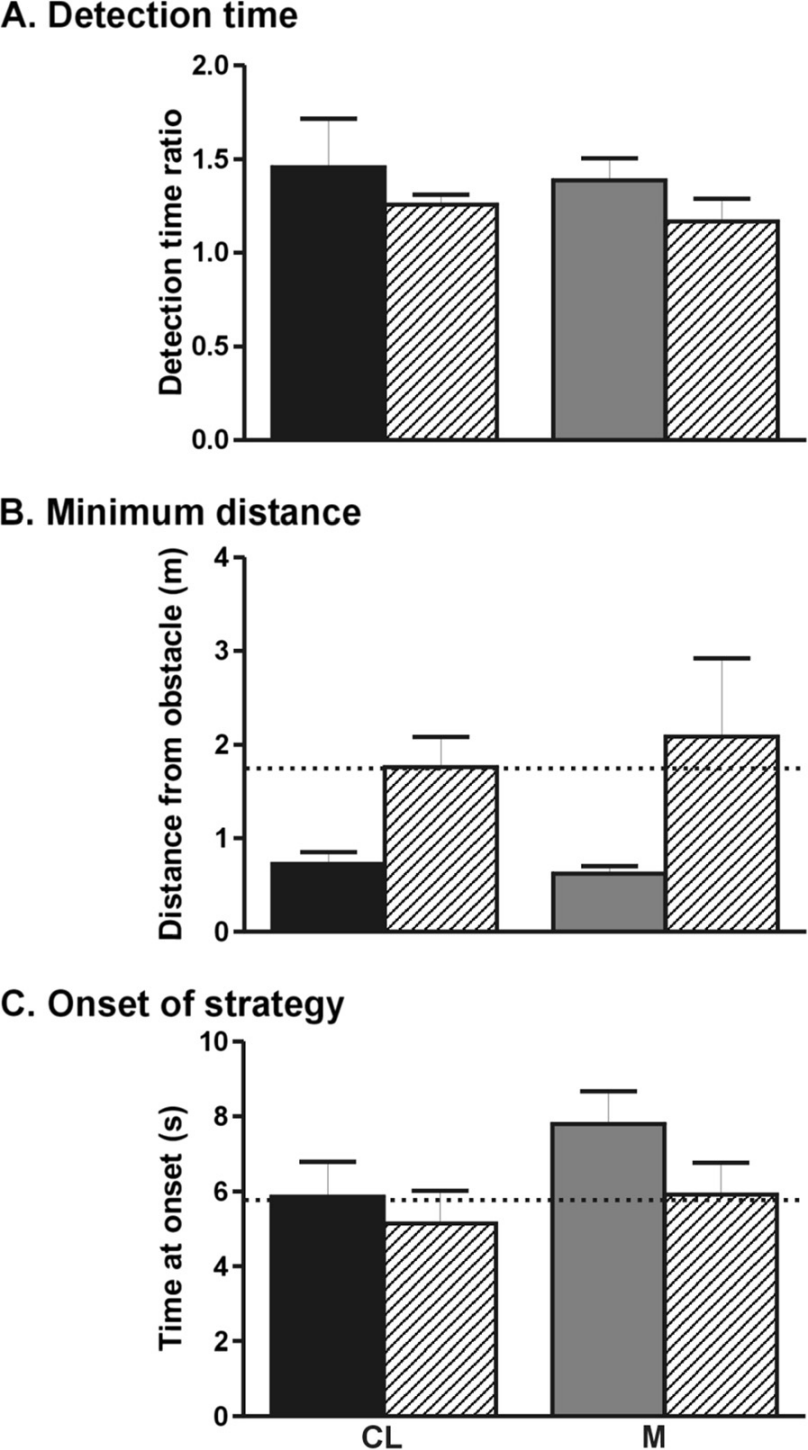
**Mean (±1SD) values for A) obstacle detection times [perceptuo-motor task], B) minimum distances and C) onset time of avoidance strategy for the colliders (solid bars) and non-colliders (lined bars) [locomotor task] are represented for the contralesional (CL) and middle obstacle (M).** Mean values across all participants for the ipsilesional obstacle approach are represented by the dotted line to provide a reference value.

To understand the factors that differentiate participants who collided from those who did not collide in the locomotor task, their performances were examined and qualitatively compared for the contralesional and middle approaches. A statistical approach was not feasible due to the small number of participants in each group. In the perceptuo-motor task, detection times for the contralesional and middle obstacles, expressed as a ratio of the ipsilesional obstacle detection time, revealed that colliders with contralesional and middle obstacles took longer to detect the obstacles compared to non-colliders (Figure 4). In the locomotor task, colliders maintained smaller minimum distances from the obstacles and initiated their avoidance strategies later compared to non-colliders for the contralesional and middle obstacles. All participants showed a preference to deviate their trajectories to the ipsilesional side, with no clear differences between the colliders (Contralesional obstacle: 83%; Middle obstacle: 78%) compared to the non-colliders (Contralesional obstacle: 71%; Middle obstacle: 91%). Note that for ipsilesional approaches and for control trials where no obstacles were moving, participants veered ipsilaterally in 86% and 74% of the trials, respectively.

Colliders and non-colliders showed similar results on the Bells, Line Bisection and Trail Making B tests (Table 2). However, colliders with the contralesional obstacles walked faster (0.56 ± 0.08 m/s) than non-colliders under the same condition (0.31 ± 0.08 m/s), as well as in control trials. No speed differences between middle obstacle colliders and non-colliders were observed during the middle obstacle approach and control trials.

**Table 2:** Characteristics of colliders and non colliders for the contralesional (CL) and middle (M) obstacle approaches

	**CL Obstacle**		**M Obstacle**	
	**Colliders**	**Non-Colliders**	**Colliders**	**Non-Colliders**
	**(n = 5)**	**(n = 7)**	**(n = 8)**	**(n = 4)**
Bells test	6 (3.1)	4.5 (1.8)	5.4 (2.0)	4.7 ( 3.1)
Line bisection	1.2 (0.5)	1.8 (0.5)	1.4 (0.5)	1.9 (0.5)
Trail Making B	159.2 (32.8)	189.7 (31.2)	173.3 (28.4)	184.5 (40.5)
CMMSA (Leg)	5.2 (0.4)	4.3 (0.5)	4.8 (0.9)	4.5 (0.5)
CMMSA (Foot)	4.0 (0.7)	3.0 (0)	3.6 (0.7)	3.0 (0)
Walking speed*	0.56 (0.08)	0.31 (0.08)	0.41 (0.08)	0.44 (0.13)
Walking speed for control trial	0.51 (0.12)	0.28 (0.19)	0.37 (0.20)	0.38 (0.23)

Mean values (one standard deviation) are presented. In the Bells test, the values reflect the average number of omissions. In the Line Bisection Test, the values are the error (deviation from the midpoint) in cm. For the Trail Making B, the values indicate the time taken to complete the test, in seconds. CMMSA, Chedoke McMaster Stroke assessment level of motor recovery for the leg and foot components (/7). *Walking speed during the specified obstacle condition in meters/second.

Finally, while the contralesional colliders tended to present a better motor recovery of the paretic leg and foot compared to contralesional non-colliders (mean difference of 1 unit on the Chedoke McMaster Stroke Assessment), the head-on colliders and non-colliders did not show much difference (See Table 2).

### Relationship between perceptuo-motor and walking performances

No significant associations were observed between detection times on the perceptuo-motor task and the participants’ performance on the locomotor task, as measured by minimum distances maintained from the obstacle, percent collisions and onset of trajectory deviation (p > 0.57). Performances on the perceptuo-motor and locomotor tasks did not correlate with the results on the Line Bisection Test, Bells Tests and Trail Making B (p > 0.5). Walking speed during the trials was not related to percent collisions but it was, however, negatively associated with the minimum distance for contralesional (r = -0.761, p = 0.004) and ipsilesional obstacles (r = -0.878, p < 0.0001). Smaller minimum distances were associated with larger percentage of collisions for contralesional and middle obstacles (Contralesional: r = -0.6366, p = 0.013; Middle: r = -0.622, p = 0.0155).

## Discussion

Previous navigation studies involving persons with VSN have aimed at understanding trajectories of walking [11,28,29], object recognition [30,31] and collision with static objects present on the side of the walking path [12,29]. A significant body of literature is also concerned with computer based navigation tasks, where the challenges of locomotion itself are not present [20,30,32]. The present study adds to previous knowledge by addressing a functional task commonly encountered in daily life using a locomotor-specific evaluation and by investigating the perceptuo-motor and locomotor factors affecting obstacle avoidance abilities. Our results demonstrate, for the first time, that persons with VSN are at greater risk of colliding with moving obstacles approaching contralesionally and from straight ahead, as opposed to obstacles approaching ipsilesionally. Colliders, while displaying a similar severity of neglect on clinical assessments compared to non colliders, take longer to identify approaching obstacles and display altered steering behaviours. The implication of such findings as well as the contribution of perceptual and locomotor factors are discussed below.

### VSN is associated withhigh rates of collisions with moving obstacles

One of the most striking findings of this study is that up to 67% of participants (8 out of 12 participants) collided with either or both the contralesional and the head on obstacle, with collisions occurring in almost 1 out of 2 trials in some of the participants. While persons with VSN are reported to bump into stationary objects [12,33], present collision rates with moving obstacles cannot be compared with previous studies given that collision rates are typically not reported. These high collision ratesmaycompromise safety while walking in community environments where moving obstacles are present. Limited community ambulation, in return, may further delay the recovery of independent walking [10] and reduce quality of life [34]. These observations highlight the importance of addressing obstacle avoidance abilities in persons with VSN.

### Interaction of perceptual and locomotor factors

The perceptual deficits in our participants were evident through larger detection times and subsequent delays in onset of avoidance strategy for the contralesional and middle obstacles. These variables also differentiated the colliders from the non-colliders. Since the joystick was held with the non-paretichand, the results were not biased by the presence of any upper-extremity motor impairment. Moreover, due to the task being a simple joystick-button click, we believe that the handedness would not invalidate the results. Similar to other studies in VSN [35,36], a gradient of increasing detection times was observed from the ipsilesional to the contralateral visual field. Minimum distances from the obstacles maintained by participants in the present study were also smaller for contralesional and middle approaches, suggesting that their ‘personal space’, defined as the perceived safe distance an individual maintains from another object/person while walking [24,37], is contracted on the contralesional side. Other possible explanations include an altered internal representation of space that is compressed towards the ipsilesional side [38], an altered sense of position with respect to objects (egocentric coordinates) located in the neglected field [39] and an ipsilesional shift of the subjective midline [11] which could cause the contralesional and the middle obstacle to remain unattended in the contralesional field.

Healthy young and elderly individuals [40], and individuals with stroke but no VSN [41], tested on a similar obstacle-avoidance paradigm, were shown to increase their ‘safety margins’ when additional attentional challenges were introduced. Older adults were also shown to slow down their gait when confronted with moving obstacles [40]. Conversely, VSN participants in the present study maintained smaller distances frommiddle and contralesional obstacles. They also maintained walking speeds similar to that adopted during the control trials where no obstacles were approaching. This absence of an adaptive response to a perceived threat is consistent with an attentional-perceptual disorder that is characteristic of VSN [3,4].

Although no direct relationships were observed between collision rates and gait speed, faster walkers maintained smaller minimum distances compared to slower walkers for the diagonally approaching obstacles. Furthermore, a qualitative comparison of colliders vs. non-colliders revealed that for the contralesional approach, colliders displayed faster walking speeds and higher level of lower-extremity motor recovery. It is interesting to note that these observations contrast with the common presumption that persons with slower walking speeds or poorer motor recovery present with a compromised walking capacity and should be at higher risk of collisions. We hypothesize that slow walking may have served as a protection by allowing diagonal obstacles passing in front of the participants, therefore preventing a collision. Slow walking speed, however, was not a ‘strategy’ or context-specific adaptation adopted by the non-colliders since their speeds were similar in the control trials. The unintentional protection offered by the fixed-speed obstacle to the slower walkers can be viewed as a limitation of the experimental design. We predict that greater collision rates may have been observed for the contralesionally approaching obstacles, had the obstacle speeds matched the walking speeds. Also, this protective effect cannot operate for middle obstacles where directional changes of the walking trajectory are required to avoid collision.

Given the absence of a comparison group of non-VSN stroke participants, one may debate whether the altered perceptuo-motor and locomotor strategies observed in the present study are attributed to VSN, or to stroke-related sensorimotor deficits. In another study from our laboratory (Aravind. G, Lamontagne. A: A virtual reality based navigation task to unveil obstacle avoidance performance in individuals with visuospatial neglect, In preparation) [41], participants with VSN were evaluated on a joystick-driven obstacle avoidance task, using their non-paretic hand to manipulate the joystick while seated. In such context that minimized postural and locomotor demands, participants demonstrated collisions with contralesional and middle obstacles, as in the locomotor task described in this study. This observation supports the hypothesis that attentional-perceptual deficits of VSN influence obstacle avoidance abilities. Rates of collision in the joystick-driven task (21% to 26%), however, were smaller than those observed during walking. This may be due to the influence of stroke-related sensorimotor impairments on locomotion and defective sensorimotor integration processes (for a review [42]), as well as to the increased complexity of the locomotor task that results in VSN becoming more apparent [43-45]. Therefore, the additional burden of locomotion may make the task more complex, increasing the rate of collisions.

Additionally, Darekar et al. [41], using a similar paradigm with obstacles approaching from the middle, ipsilesional and contralesional directions, have shown that individuals with stroke without VSN demonstrated) no collisions with any of the three obstacles and ii) a tendency to maintain larger distances from obstacles compared to healthy controls, a behaviour that is contrasting to our participants with VSN. Thus the presence of sensorimotor deficits post-stroke alone cannot explain the tendency to collide with moving objects.

### Need for task-specific assessments of ambulation abilities

Contrary to our expectations, no associations were observed between the participants’ performance on the locomotor task and that on the perceptuo-motor task. This is somewhat surprising given that colliders performed worse, on average, compared to non-colliders on the perceptuo-motor task. We suggest that the participants’ locomotor and perceptuo-motor abilities have interacted in generating altered obstacles avoidance strategies, a hypothesis that may be further verified in a larger sample of participants. The perceptuo-motor task also differed from the locomotor tasks in that the participants were seated and responded with a single-alternative button press, facing none of the complex locomotor demands. Persons with VSN may prioritize the limited attentional resources to the control of walking, hence compromising the attention diverted to extrinsic stimuli [11]. Responses on the perceptuo-motor task may not entirely reflect perception while walking.

A lack of relationship was also observed between the participants’ performance on the laboratory tasks and clinical scores of VSN, which support previous observations that paper-pencil tests fail to predict performance on visually-guided functional tasks [21,23]. These clinical tests are limited to near space [46] and static visual stimuli [22] and they lose sensitivity for milder cases [47] with many of them being originally designed for visual attention and cognitive assessments rather than VSN [48]. Therefore it is essential to carry out a functional, task-specific assessment to appreciate the recovery achieved and the challenges faced by the individuals. The obstacle avoidance behaviours observed in the VE are closely related to the real-world strategies [24,49]. Therefore, the performance of individuals with VSN in our study can provide information regarding their safety during community ambulation, lending support to the external validity of our findings. This experimental paradigm can be used to assess and potentially train individuals with neglect after stroke to avoid moving obstacles while walking.

## Conclusions

Persons with post-stroke VSN show a delayed perception and experience collisions with obstacles approaching from the contralesional side and from straight ahead, as opposed to obstacle approaching from the ipsilesional side. The longer obstacle detection times in the colliders compared to non-colliders suggest that attentional-perceptual deficits, along with sensorimotor impairments and altered sensorimotor integration processes due to the stroke, influence obstacle avoidance strategies and lead to collisions. The failure of clinical tests of VSN to predict the participants’ performance on the obstacle avoidance task emphasizes the need for a task-specific assessment of ambulation abilities.

## Abbreviations

VSN: Visuospatial neglect; VE: Virtual environment; CT: Computerised Tomography; MRI: Magnetic Resonance Imaging; MVPT: Motor free visual perceptual test; SD: Standard deviation; HMD: Helmet mounted display; ANOVA: Analysis of variance.

## Competing interests

The authors declare that they have no competing interests.

## Authors’ contributions

GA conceived, collected and carried out the experiments. She also carried out the data reduction, analyses of the data and drafting of the manuscript. AL contributed to the design of the study, data analysis, interpretation of results and revision of the manuscript. Both authors read and approved the final manuscript.
